# Intracellular *Eimeria bovis* macromeront formation induces bystander cell accumulation and TNT formation

**DOI:** 10.3389/fcimb.2025.1665269

**Published:** 2025-09-26

**Authors:** Jobst Fischer, Lara Sous, Zahady D. Velásquez, Carlos Hermosilla, Anja Taubert

**Affiliations:** Institute of Parasitology, Biomedical Research Center Seltersberg, Justus Liebig University of Giessen, Giessen, Germany

**Keywords:** apicomplexan parasite, parasite host-cell interactions, *Eimeria bovis*, tunnelling nanotubes, mitochondrial transfer, endothelial cells

## Abstract

**Introduction:**

*Eimeria bovis* first merogony is an intracellular process (~ 3 weeks) resulting in the formation of large macromeronts (≤ 400 μm) containing up to 140,000 merozoites I, each. The production of merozoites I poses critical metabolic stress on bovine endothelial host cells, leading to mitochondrial dysregulation and premature senescence. In this context, an accumulation of non-infected bystander cells (BCs) around *E. bovis* macromeront-carrying host cells (MCHCs), eventually supporting MCHCs, was observed.

**Methods:**

BC accumulation was quantified by 3D confocal microscopy. A meront-transfer-system was established to evaluate the supportive BC capacity of different cell types. Since healthy cells might support stressed cells by transferring cargo like mitochondria via TNTs, we studied if E. bovis infection affected cellular TNT formation. By utilizing the meront-transfer-system, recipient non-infected BCs were pre-treated with inhibitor of TNT formation (cytochalasin B) and the effect on *E. bovis* development was estimated in BC-MCHC-cocultures. To study the transfer of mitochondria via TNTs, non-infected and E. bovis-infected cells where stained with respective dyes and cargo transfer was illustrated.

**Results:**

In *E. bovis*-infected cell layers, an increase of BCs at all sides of MCHCs was stated, thereby correlating with meront sizes and maturation. When using different cell types as BCs, we showed that macromeront development was best supported by human endothelial cells, followed by human fibroblasts and bovine endothelial cells. Overall, TNT numbers were increased in *E. bovis*-infected cell layers. The relevance of TNTs for parasite development was underlined by selective BC cytochalasin B treatments, which blocked both TNT formation and merozoite I production. Given that TNT-based transfer may improve the energetic status of E. bovis-infected cells, we observed bidirectional mitochondrial transfer between non-infected and *E. bovis*-infected cells, thereby potentially helping to restore the energetic status of the infected host cell.

**Discussion:**

Bystander cell-based TNT-mediated mitochondria transfer may evidence a new mechanism of parasite-induced host cell modulation, aiding MCHCs to support parasite proliferation.

## Introduction

*Eimeria bovis* is a globally occurring, obligate intracellular apicomplexan parasite of cattle (*Bos* spp.). *E. bovis* infections, known as bovine eimeriosis (coccidiosis), often remain subclinical, but may also cause severe haemorrhagic typhlocolitis in calves. In both scenarios, significant economic losses are the consequence. Hence, statistical surveys calculated *Eimeria* spp.-related losses by 8-9% of annual revenue, thereby only considering clinical cases (Lassen and Ostergaard, 2012). Currently, drug interventions are limited to a few metaphylactic drugs, for which resistance has already been described in ovine *Eimeria* spp. (Odden et al., 2018).

Within the host, *E. bovis* undergoes different phases of asexual (i. e. merogonies I and II) and sexual (gamogony) replication (Hermosilla et al., 2002). During first merogony, *E. bovis* multiplies within lymphatic endothelial cells of intestinal villi. In contrast to most other bovine *Eimeria* species, *E. bovis* merogony I results the formation of large intracellular macromeronts with a size of up to 400 μm, containing > 140,000 merozoites I per meront (Hammond et al., 1946, 1966). For this massive and long-lasting (~ 3 weeks) intracellular parasite proliferation, *E. bovis* is in considerable need of energy and building blocks and vastly manipulates its host cell to satisfy its metabolic and structural requirements (Hermosilla et al., 2008; Taubert et al., 2010). Hence, lipid and cholesterol metabolism, glycolysis, innate immune responses, apoptosis and cytoskeleton are significantly affected in host cells during *E. bovis* first merogony (Taubert et al., 2006; Hermosilla et al., 2008, Hamid et al., 2014, 2015; Taubert et al., 2018; Velásquez et al., 2021b; Silva et al., 2022). Moreover, *E. bovis* infection of primary bovine endothelial cells fosters host cellular cell cycle arrest at G_1_ phase, finally inducing both premature senescence and mitochondrial dysfunction (Velásquez et al., 2021b), with all these findings reflecting a critical parasite-driven metabolic and energetic host cell status. Nevertheless, macromeront-carrying host cells survive these extreme demands and support parasite development until merozoite I release (Hermosilla et al., 2012). However, it seems unlikely that a single (host) cell can fulfil all proliferative demands of *E. bovis* during macromeront formation and withstands the ongoing metabolic and energetic stress without the help of other cells. In line, macromeront-carrying host cells (MCHCs) were recently illustrated to be closely surrounded by several non-infected bystander cells (Velásquez et al., 2021b) seemingly encasing infected host cells. In other context, it is well-documented that injured or senescent cells are supported and revitalised by healthy cells via tunnelling nanotubes (TNTs)-based mitochondria donation (Yasuda et al., 2010; Liu et al., 2014; Walters and Cox, 2021). TNTs are tubular plasma membrane bridge structures with a diameter of 50–1500 nm used to connect cells. The length of TNT connections ranges from a few to hundreds of micrometres. While all TNT phenotypes contain filamentous F-actin as a backbone, they might also comprise microtubules or specific myosin motor molecules (Ljubojevic et al., 2021). In general, a broad range of cell types are able to form TNTs including endothelial cells allowing for the exchange of a wide range of molecules and organelles (Cervantes and Zurzolo, 2021; Ljubojevic et al., 2021). As such, TNT-transferred cargo includes ions, mRNAs, peptides, proteins, lipid droplets, lysosomes, endoplasmic reticula (ER), Golgi vesicles and mitochondria, amongst others (Rustom et al., 2004; Austefjord et al., 2014; Drab et al., 2019; Jansens et al., 2020; Dagar et al., 2021). The transfer of organelles like mitochondria was demonstrated to support the metabolic recovery of recipient cells, thereby allowing them to return into a normal operating state, back from apoptotic processes (Luchetti et al., 2022). So far, it remains to be elucidated if bystander cell accumulation around MCHCs may be linked to TNT formation and organelle/molecule transfer. Thus, we here investigated the potential role of TNT formation, mitochondria transfer and bystander cell accumulation in the development of large-sized *E. bovis* macromeronts. Current findings add novel data not only on *E. bovis* but most likely also on other pathogenic ruminant macromeront-forming *Eimeria* species.

## Materials and methods

### *Eimeria bovis* (strain H) experimental infection of calves

Two weeks old male Holstein Friesian calves (*n* = 3) were purchased from a local dairy farm and kept in parasite-free conditions in stainless-steel metabolic cages (Woetho, Emmendingen, Germany) until experimental infection in an experimental large animal shelter (Institute of Parasitology, Justus Liebig University Giessen) equipped with an airlock entrance. Animals were screened for parasitic infections every 3 days. Calves were fed with milk substitute (Milkvit, Trouw Nutrition Deutschland GmbH, Burgheim Germany) and commercial concentrate (Raiffeisen Vital eG, Hamm, Germany). Water and sterilised hay were given *ad libitum*. At the age of 7–8 weeks, calves were orally infected with 3 x 10^3^ *E. bovis* sporulated oocysts [these were washed thrice in water (600×g, 15 min) before infection] (Silva et al., 2022). The current *E. bovis* strain H was initially isolated from the field in Northern Germany and since then maintained by passages in parasite-free male Holstein Frisian calves (Fiege et al., 1992). Animal health was routinely monitored during the total infection period. All animal procedures were performed according to the Justus Liebig University (JLU) Giessen Animal Care Committee guidelines, approved by the Ethic Commission for Experimental Animal Studies of the State of Hesse (Regierungspräsidium Giessen, GI 18/10 No V1/2022, JLU-No. 0001-V) and in accordance to the current German Animal Protection Laws.

### *Eimeria bovis* oocyst isolation

Calves were orally infected, as described above, and subsequently, the collection, sporulation, and storage of oocysts was performed as previously described by Taubert et al. (2018). For excystation, sporulated oocysts were suspended in sterile 0.022 M _L_-cysteine-hydrochloride (Sigma Aldrich, St. Louis, MO, USA),/0.2 M NaHCO_3_ (Sigma Aldrich, St. Louis, MO, USA) aqueous solution and incubated for 16–20 h in a 100% CO_2_ atmosphere at 37°C. Then, oocysts were pelleted (600 × g,15 min, 20°C) and resuspended in 1x Hank’s balanced salt solution (HBSS; Gibco, Fisher Scientific GmbH, Schwerte, Germany) containing 0.04% (w/v) trypsin (Sigma-Aldrich, St. Louis, MO, USA) and 8% (v/v) sterile-filtered (0.2 µm filter; Sarstedt, Nümbrecht, Germany) bovine bile obtained from the local abattoir. The oocysts were incubated for up to 4 h (37°C, 5% CO_2_) under constant microscopic control. Free sporozoites were washed twice (600 × g, 15 min, 20°C) in sterile medium (M199; Sigma Aldrich, St. Louis, MO, USA), passed through a 10-µm pore-size filter (pluriStrainer, PluriSelect, Leipzig, Germany, Life Science) according to López-Osorio et al. (2020), and counted in a Neubauer chamber (Karl Hecht GmbH & Co KG, Sondheim vor der Rhön, Germany). Free *E. bovis* sporozoites were used to infect primary bovine umbilical vein endothelial cells (BUVEC).

### Isolation and maintenance of primary BUVEC

Primary BUVEC were isolated from bovine umbilical cords according to Taubert et al. (2018). In short terms, umbilical cords were collected under aseptic conditions from animals born by *sectio caesarea* and endothelial cells were isolated by treatments with 0.025% collagenase type II (Worthington Biochemical Corporation, Lakewood, NJ, USA) suspended in aqueous Pucks solution [NaCl (0.8% w/v), KH_2_PO_4_ (0.015% w/v), KCl (0.04% w/v), CaCl_2_ (0.0012% w/v), Carl Roth GmbH & Co. KG, Karlsruhe, Germany, MgSO_4_·7H_2_O (0.0152% w/v), NaH_2_PO_4_ (0.039% w/v) Merck/Sigma-Aldrich St. Louis, MI, USA], which was infused into the lumen of ligated umbilical veins. After an incubation period of 20 min (37°C, 5% CO_2_), the cell suspension was collected in cell culture medium (20 ml) and supplemented with 1 ml fetal calf serum (FCS; Gibco, Fisher Scientific GmbH, Schwerte, Germany) to inactivate collagenase. After washing (350 × g,12 min, 20°C), cells were resuspended in complete endothelial cell growth medium (ECGM, Promocell, Heidelberg, Germany, supplemented with 5% FCS), seeded in 75-cm^2^ tissue plastic culture flasks (Greiner, Frickenhausen, Germany), and kept at 37°C in 5% CO_2_ atmosphere. BUVEC were continuously cultured in modified ECGM (modECGM) medium [ECGM diluted at 30% in M199 medium, supplemented with 5% FCS (Gibco, Fisher Scientific GmbH, Schwerte, Germany), 1% penicillin and streptomycin (PS, Sigma-Aldrich, St. Louis, MO, USA)] with medium changes every 2–3 days. BUVEC layers were used for infection after one to three passages *in vitro*.

### Enrichment of *Eimeria bovis* macromeront-carrying host cells

**Figure 1:**
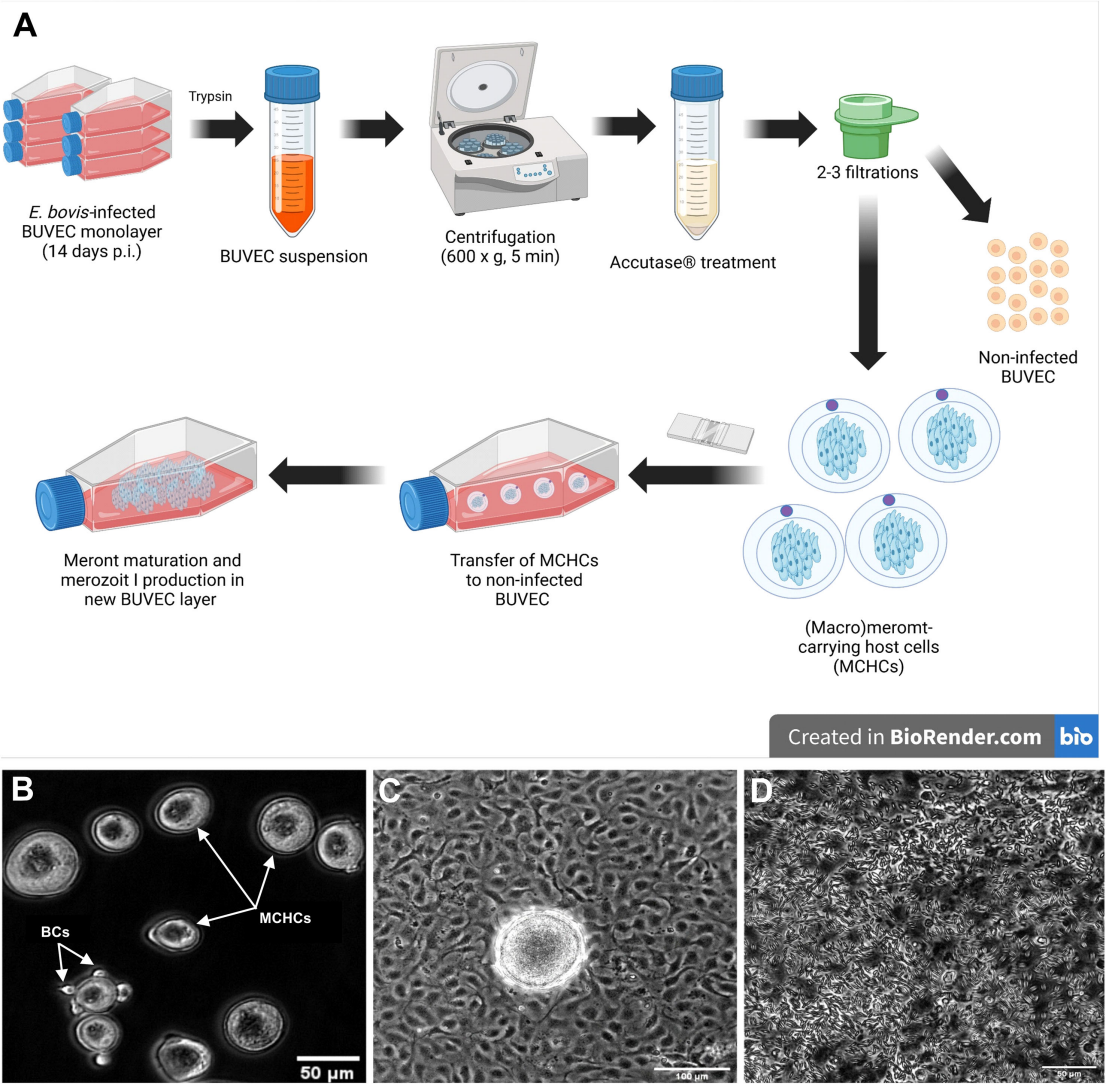
**(A)** Experimental workflow for the meront-transfer-system. **(B)** Purified and transferrable *E*. *bovis* MCHCs after enzymatic treatment and filtration. Few residual BCs (white arrow) are present in enriched MCHC suspensions. **(C)** Phase contrast illustration of MCHCs after transfer to a non-infected BUVEC layer **(D)** merozoites I release by a transferred MCHC, 10 days post transfer.

BUVEC (*n* = 3 - 4) were seeded on bovine fibronectin (1:400 in PBS, Sigma Aldrich, F1141-2MG, St. Louis, MO, USA)-coated 75-cm² tissue flasks. At 90% confluency, BUVEC were infected with *E. bovis* sporozoites (7.5 x 10^5^ - 1.5 x 10^6^ sporozoites per 75-cm² tissue flask, Greiner Bio-One GmbH, Frickenhausen, Germany). The cell culture medium was changed 24 h after parasite infection and thereafter every 2 days. On day 14 p. i., infected BUVEC were detached by trypsin treatments [0.25% (w/v) (Sigma-Aldrich, St. Louis, MO, USA), 4 ml per 75-cm² tissue flasks, 5 min, 37°C]. Trypsinization was stopped by supplementation of 8 ml modECGM. The cell suspension was transferred to a 15 ml Falcon™ tube (Thermo Fisher Scientific, Waltham, MA, USA) and sedimented (400 × g, 5 min, RT). The cell pellet was well resuspended and incubated (15–45 min) in 5 ml accutase (Accutase Cell Detachment SolutionACC-1B, Capricorn Scientific, Ebsdorfergrund, Germany) to improve cell singulation, which was monitored microscopically. After full singulation, the cell suspension was filtered by a sterile 20 μm cell strainer (pluriStrainer, PluriSelect, Leipzig, Germany, Life Science) attached to an adaptor (pluriSelect, pluriStrainer, PluriSelect, Leipzig, Germany, Life Science) deposited on a 50 mL Falcon™ tube to remove non-infected BUVEC (which passed through) and to enrich MCHCs (> 20 μm diameter) which remained on top. After thoroughly washing with modECGM, the strainer was inverted to flush-out MCHCs with modECGM (6 ml) into wells of a 6-well cell culture plate (Sarstedt AG & Co. KG, Nürnbrecht, Germany). After transfer, MCHC purification was controlled microscopically (IX81, Olympus) (**Figure 1**). If necessary, the filtration step was repeated 1–2 times until the desired MCHC purity of non-infected BUVEC/MCHC ≤ 1 was achieved.

### Image acquisition and analyses

For phase contrast and fluorescence analyses, images were taken by an inverted microscope (IX81, Olympus, Olympus Plan NA 0.25, 10x, Shinjuku City Tokyo, Japan, or Keyence, BZ-X800, Plan Fluorite 20x, NA 0.45) equipped with a digital camera (XM10, Olympus). Image analysis was carried out either by Image J (imagej.net) or by BZ-X800 Viewer software (Keyence, Neu-Isenburg, Germany). Confocal Z-Stacks and BC quantification were performed with a ReScan Confocal instrumentation (RCM 1.1 Visible, Confocal.nl) equipped with a fixed 50 µm pinhole size and combined with a Nikon Ti-2 Eclipse microscope with a motorized Z-stage (DI1500, Nikon). The RCM unit was connected to a Toptica CLE laser with the following excitations: 405/488/561/640 nm and operated by the NIS-Elements software. 3D rendering was performed in Image J with 3D Viewer plugin. Images processed by deconvolution (Adaptive algorithm LAS X Lightning tool) were obtained by a Leica SP8 (Leica Microsystems, Wetzlar, Germany) confocal microscope (at Paul-Ehrlich-Institute, Langen, Germany) and analysed with the Leica LAS X Life software.

### Visualisation of mitochondria and actin-/tubulin-based cytoskeleton

Cells were grown on glass coverslips (⊘1.5 cm) deposited in a 12-well cell culture plate (Sarstedt AG & Co. KG, Nürnbrecht, Germany) and fixed with 4% paraformaldehyde (PFA) for 20 min at RT. After fixation, the cells were washed three times with PBS and then incubated in blocking/permeabilization solution (PBS with 3% BSA, 0.1% Triton X-100, Sigma Aldrich, St. Louis, MO, USA) for 1 h at RT. Thereafter, samples were incubated with primary antibodies (see **Table 1**) diluted in blocking/permeabilization solution overnight at 4°C in a humidified chamber. After three additional washes in PBS, the samples were incubated in secondary antibody solutions (see **Table 1**) for 30 min at RT in darkness. Actin was stained with phalloidin (00042, Biotium, 6.7 µl/well for 30 min) during incubation with secondary antibodies. Cell nuclei were stained with 4′, 6-diamidino-2-phenylindole (DAPI) present in mounting medium (495952, Fluoromount G, Invitrogen, Carlsbad, CA, USA). In additional experiments, live and fixed cells were stained with MitoView™ Green (70054, 50 nM, 30 min, Biotium, Fremont, CA, USA) or MitoTracker™ RedCMXRos (M7512, 33 nM, 30 min, Invitrogen™).

**Table 1:** Primary and secondary antibodies used in the study.

Antibodies	Company	RRID	Cat. Nr.	Origin	Dilution
Primary					
Anti-*E. bovis*	In-house	−	−	Bovine	1:1000
Anti-AIF	Abcam™	AB_726995	ab32516	Rabbit	1:100
Anti-α tubulin	Invitrogen™	AB_221538	A11126	Mouse	1:500
Secondary					
AlexaFluor 647	ThermoFisher	AB_2535804	A21235	Goat	1:500
AlexaFluor 594	ThermoFisher	AB_2556545	R37117	Goat	1:500
Anti-bovine FITC	Invitrogen™	AB_2535983	A2441	Goat	1:500

The table shows the antibodies, supplier information, RRIDs, catalog numbers, host origin, and working dilutions applied for immunofluorescence experiments.

### Bystander cell quantification

BUVEC (*n* = 3) were seeded on 12-well cell culture plates (Sarstedt AG & Co. KG, Nürnbrecht, Germany) containing fibronectin-coated [0.0025% (v/v), Sigma Aldrich, St. Louis, USA] glass coverslips (⊘1.5 cm). At 90% confluency, BUVEC were infected with *E. bovis* sporozoites (7.5 x 10^5^/well). At days 8, 12, 15, 18, and 22 p. i., BUVEC were fixed with 4% PFA for 20 min at RT. After three washings in PBS, coverslips were mounted on cell samples with a drop of Fluoromount-G™ containing DAPI. BC quantification was performed by a confocal microscope via screening the Z-axis in 200x magnification at an excitation of 405 nm, counting the visible DAPI-stained nuclei surrounding the MCHCs. BCs in lateral, ventral and dorsal position of the *E. bovis*-infected host cell were counted. For controls, neighbouring cells in non-infected BUVEC layers (*n* = 3) where counted to exclude a cell density-based cell accumulation effect reflecting a normal growth behaviour of BUVECs being cultured over a long time (i. e. ≈ 3 weeks).

### Transfer of MCHCs to different non-infected cell cultures

To study the role of BC origin, enriched MCHCs were transferred to cultures of different primary cell types, serving as BC source. Therefore, BUVEC, human umbilical vein endothelial cells (HUVEC; Gibco™ Thermo Fisher Scientific, Waltham, MA, USA) and human foreskin fibroblasts (HFF; Sigma Aldrich, St. Louis, MO, USA) were used in co-culture systems and tested for their support of *E. bovis*-macromeront development. First, BUVEC (*n* = 3 - 4) were seeded and infected with *E. bovis* sporozoites in T75-cm^2^ tissue flasks (Greiner Bio-One GmbH, Frickenhausen, Germany). At 14 days p. i. MCHCs from pooled BUVEC where isolated and transferred (1.5 x 10^3^ meronts/well) to non-infected cell layers of BUVEC, HUVEC or HFF (all: *n* = 4) which had previously been seeded in 12-well cell culture plates coated with fibronectin (5 x 10^4^ cells/well). For controls, an equal number of MCHCs were transferred into cell-free wells. Every third to fifth day, the supernatants were collected and merozoite I numbers were counted in a Neubauer chamber covering up to 26 days p. i. Besides merozoite I numbers, macromeront sizes (µm^2^) were assessed microscopically at day 22 p. i. via Image J.

### TNT imaging and quantification

BUVEC (*n* = 5) were seeded on fibronectin-coated 12-well cell culture plates (3 x 10^5^ cells/well; Greiner) at three technical replicates, each. After 48 h, subconfluent (30-40% confluency) cell layers were used for TNT quantification according to Tishchenko et al. (2020). For each BUVEC isolate, 9–15 microscopic images were randomly taken using a phase contrast microscope (100x magnification) and analysed for TNT formation by ImageJ software using Olympus Viewer and cell counter plugin. Here, exclusively vital, singulated BUVEC were analysed and all membrane protrusions and connecting membrane bridges fitting into the settings of 70–4000 nm diameter were counted. Moreover, to prove the TNT nature of these subcellular structures, BUVEC were fixed with 4% PFA, and thereafter stained for actin, tubulin and mitochondria (**Table 1**) as described above.

For the quantification of TNT formation in *E. bovis*-infected BUVEC cell layers, BUVEC (*n* = 4) were cultured on bovine fibronectin-coated 6-well cell culture plates at three technical replicates, each. BUVEC layers were infected with *E. bovis* sporozoites (3 x 10^5^ per well, obtaining an initial infection rate of 10-20%), or left uninfected for negative controls. The cell culture medium was changed 24 h after parasite infection and thereafter every 2–3 days. At days 3 or 4, 7 or 8, 11 or 12, 17 and 24 p. i., infected cells and non-infected controls were detached by trypsinization [1 ml 0.25% (w/v) trypsin, 0.05% EDTA (w/v), 5 min], which was stopped by addition of 2 ml of modECGM. Cell suspensions were pelleted (400 x g, 5 min, RT). The cell pellet was very well resuspended in 1 ml modECGM for cell isolation. Subsequently, infected and non-infected BUVEC (3 x 10^5^/well) were cultured for 48 h on fibronectin-coated 12-well cell culture plates (three technical replicates) and then analysed for TNT formation as described above by analysing nine random microscopic images/condition. Here, TNT formation was assessed in both, confluent *E. bovis*-infected BUVEC layers (containing infected and non-infected cells) and in single outgrowing *E. bovis*-infected host cells.

Since *E. bovis* infection of BUVEC cultures may also affect TNT formation via the release of molecules and organelles into the medium, we also tested infection-conditioned medium (ICM) for its effect on TNT formation. Therefore, cell supernatants were collected throughout *in vitro* infection (at days 1 – 4, 4 – 7, 8 – 11, 11 – 15, 15–19 p. i.) from *E. bovis*-infected BUVEC (*n* = 4) grown in T25-cm^2^ tissue flasks (Greiner Bio-One GmbH, Frickenhausen, Germany). In parallel, cell supernatants from identical non-infected BUVEC isolates (*n* = 4) were collected for controls. After collection, supernatants were sterile filtered (0.2 µm filter; Sarstedt, Nümbrecht, Germany), frozen and stored (-20°C). For analysing the effects of ICM treatments, BUVEC isolates (*n* = 4, each at three technical replicates) were seeded on fibronectin-coated 12-well cell culture plates (3 x 10^5^ cells/well; Greiner). One day after seeding, cells were treated with ICM supplemented by control medium [50% (v/v) with modECGM] for 24 h and microscopic images (at least nine images per condition) were randomly taken. TNT formation was assessed as described above.

### Imaging of TNT-based mitochondria transfer

To image the kinetics of TNT-based mitochondria transfer in *E. bovis*-infected host cell layers, subconfluent MitoView Green (100 nM, 15 min, Fermont, CA, USA) -stained *E. bovis-*infected BUVEC (4 days p. i.) were monitored by live cell microscopy for 25.5 min using an inverted microscope (BZ-X800, Keyence) equipped with a top-stage incubator (okolab™, Ottaviano, NA, Italy) applying adequate culture conditions (37°C, 5% CO_2_). Moreover, using the meront-transfer-system (see above), the kinetic of TNT formation was illustrated by time lapse-based monitoring, covering a time period of 11 h of TNT formation in a single MCHC (15 days p. i.) using an inverse microscope (Nikon Ti-2 Eclipse) supplemented with a ReScan Confocal instrumentation (RCM 1.1 Visible, Confocal.nl) and a top-stage incubator (okolab™). Finally, to image the direction of TNT-based mitochondria exchange between infected and non-infected cells, enriched MCHCs were labelled for mitochondria with the live dye MitoTracker Red (50nm, 30 min, Thermo Fisher Scientific, Waltham, MA, USA) and transferred to subconfluent non-infected BUVEC previously labelled for mitochondria with the live dye MitoView Green (100 nM, 15 min, Biotium)). After one day of co-culture, cells were fixed (4% PFA) and analysed by fluorescence microscopy (IX81, Olympus). Additionally, to image the direction of TNT-based mitochondria exchange between MCHC and non-infected cells, subconfluent *E. bovis* layer were labelled at day 14 p. i. for mitochondria with the live dye MitoTracker Red (50nm, 30 min, Thermo Fisher Scientific, Waltham, MA, USA). One day after staining the layer was screened for TNT-based transfer between MCHC (15 days p. i.) and non-infected BUVEC by time lapse analyses (45 min) using a BZ-X800 microscope (Keyence) equipped with a top-stage incubator (okolab™, Ottaviano, NA, Italy) applying adequate culture conditions (37°C, 5% CO_2_).

### Inhibition of TNT formation

TNT formation was recently described to be selectively inhibited by low-dose cytochalasin B treatments (Bukoreshtliev et al., 2009). Therefore, BUVEC (3.5 x 10^5^/well, *n* = 4) were seeded on fibronectin-coated 12-well plates at three technical replicates, each. One day after seeding, cells were treated with cytochalasin B [350 nM, Thermo Fisher Scientific (228090010), Waltham, MA, USA] for 24 h. These compound concentration had previously been identified as non-toxic via cell viability tests. Microscopic images (at least nine images per condition) for the assessment of TNT formation were randomly taken. TNTs were quantified as described above.

To assess the effect of TNT inhibition in BCs for *E. bovis* macromeront development, non-infected BUVEC (*n* = 4) were seeded on bovine fibronectin-coated 24-well plates and treated for 24 h with cytochalasin B (350 nM) or plain modECGM (control). After thorough washing in PBS and medium replacement, enriched MCHCs (14 days p. i.) were transferred to pre-treated BUVEC layers, functioning as BCs. Free-released merozoites I present in cell supernatants were quantified in a Neubauer chamber from one-day post transfer onwards.

### Statistical analysis

All data were expressed as arithmetic mean with standard deviation. Statistical comparisons between non-infected and infected, present and non-present BCs or non-treated and treated cells used unpaired, non-parametric Mann-Whitney U-tests and Kolmogorov-Smirnov tests with the help of GraphPad Prism^®^10 software applying a significance level of 5%.

## Results

### Accumulation of non-infected BCs around *Eimeria bovis*-infected host cells increases with ongoing parasite development

**Figure 2:**
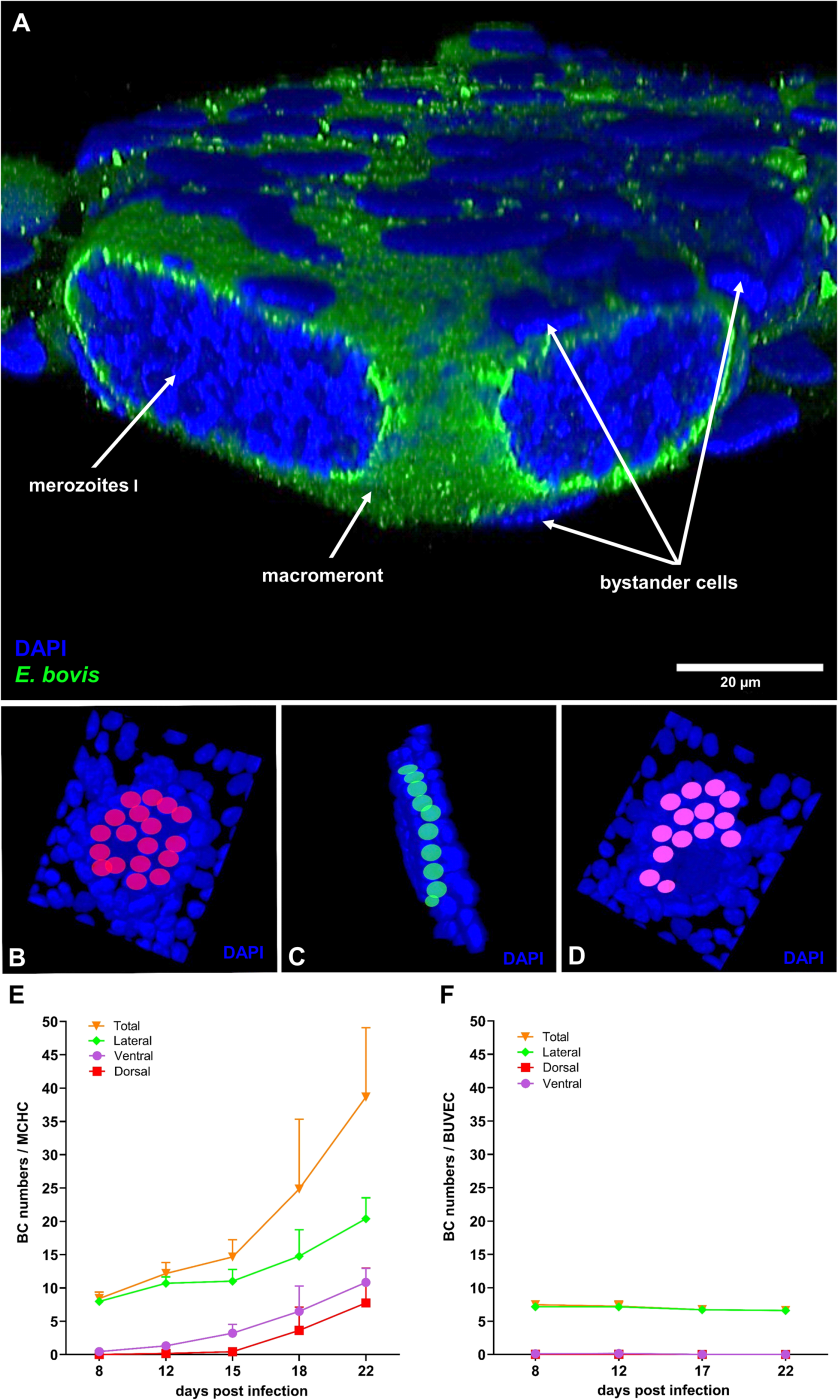
Bystander cell accumulation around *E*. *bovis* macromeront-carrying host cells. **(A)** 3D z-Stack of *Eimeria bovis* macromeront-carrying host (MCHC) cell with bystander cells (BC) in juxtaposition **(B–D)** Exemplary procedure of BC quantification. Dorsal, lateral and ventral BCs are indicated in red, green and purple, respectively. **(E)** Quantification of BC accumulation around MCHCs throughout macromeront *in vitro* development. BC quantity increases over the time of 8–22 days p. (i) Arithmetic mean and standard deviation of each three biological replicates. **(F)** Quantification of BC accumulation around non-infected control BUVECs. Dorsal, ventral, lateral BC number did not change significantly over 22 days.

During first merogony, *E. bovis* forms macromeronts exclusively within bovine endothelial cells of the ileum, which may contain > 140.000 merozoites I. It seems unlikely that this energy- and building block-demanding process of offspring formation is accomplished by one single host cell without support from the extracellular environment. In this context, Silva et al. (2022) recently illustrated MCHCs being surrounded by an enhanced number of non-infected bystander cells, but did not validate this observation by quantitative data. To quantify BC accumulation around MCHCs, DAPI-stained *E. bovis*-infected BUVEC layers containing both infected and non-infected cells (10 – 20% infection rate) were here analysed by 3D confocal microscopy throughout merogony I (days 8–22 p. i.). By counting neighbouring cells present at all sides of *E. bovis*-infected cells (for the experimental procedure refer to **Figures 2B–D**) and of non-infected cells in control cell layers, 243 MCHCs and 166 non-infected cells, respectively, from three biological replicates were here analysed for each time point. In line to Silva et al. (2022), MCHCs were here confirmed to be closely surrounded by non-infected BCs in *E. bovis*-infected BUVEC layers (**Figure 2A**). Hence, BCs in juxtaposition were not only found at lateral positions, but also on top and at ventral sides of infected host cells (**Figure 2E**). Overall, BC numbers increased with ongoing macromeront development, thereby correlating with the size and maturation of this parasite developmental stage. Thus, at the latest time point of the experiment (i. e. 22 days p. i.), the highest number of BCs in juxtaposition to MCHCs were found by showing a mean number of 19.8 BCs on lateral sides, 10.7 BCs on dorsal sides, and 5.7 BCs on ventral sides (**Figure 2E**). In comparison, non-infected BUVEC did not cause BC accumulation during the experimental period. Hence, the average of total BC numbers (≈ 7.04 BCs) in control conditions remained constant. As expected, BCs in dorsal or ventral position were not observed in control conditions (**Figure 2F**).

### Establishment of a (macro)meront-transfer-system

**Figure 3:**
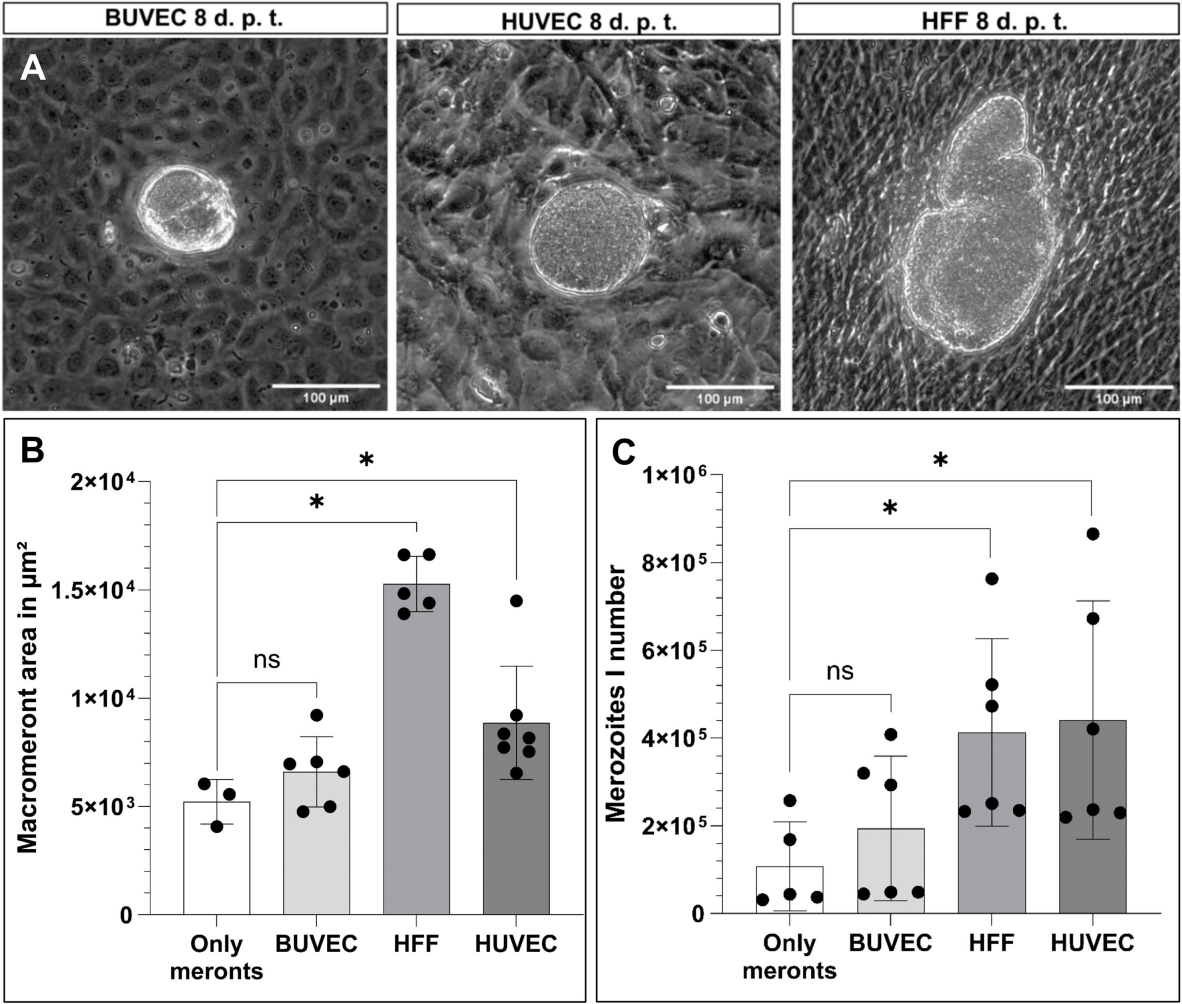
Suitability of different primary cell lines to support macromeront maturation up to merozoite I release. **(A)** Exemplary illustration of MCHC transferred to different cell lines (BUVEC, HUBEC, HFF), 8 days post transfer (= (d) p. t.). **(B)** Macromeront area measurements and **(C)** merozoite I quantification. Arithmetic mean and standard deviation of at least three biological replicates; ns, not significant; *p ≤ 0.05.

In order to distinguish MCHC- from BC-derived reactions, a (macro)meront-transfer-system was here established. Therefore, MCHCs were enriched from *E. bovis*-infected BUVEC cultures at different time points post infection (12, 14, 16, 19 days p. i.) and purified via consecutive filtering steps (for detailed experimental procedure, please see **Figure 1A**). Purified MCHCs (**Figure 1B**) were then tested for their viability, reintegration capacity and ongoing maturation after transfer to non-infected cultures. Considering infection time points of purification, meronts of 14 days p. i. were best purified, based on their differences in size compared to non-infected cells from the same cell layer. Overall, transferred MCHCs reintegrated well into the recipient cell layers (**Figure 1C**), thereby proving their well-preserved viability after isolation. At 4–10 days post transfer, equivalent to 18–25 days p. i., viable merozoites I were released into cell supernatants (**Figure 1D**), confirming adequate parasite development. Accordingly, merozoites I from transferred meronts showed normal size and motility when compared to those from non-transferred controls. Of note, effective macromeront formation within MCHCs obviously depended on the presence of neighbour cells, since pure MCHCs survived but showed lower merozoite I production after transfer into cell-free cell culture dishes (**Figure 3C**). Concerning purification efficiency, a rather high loss of MCHCs was stated applying the current isolation protocol, since up to 23% of MCHCs from donor cultures were obtained at 14 days p. i. However, for the sake of MCHC purity (99.42%), the rather low recovery rate was considered acceptable.

### Different cell types differ on quantitative level in their support of *Eimeria bovis* macromeront development

In the bovine host, macromeronts of the parasite *E. bovis* exclusively develop in endothelial cells of the central lymph vessels of small intestinal villi. Consequently, infected endothelial cells *in vivo* are physiologically not only closely surrounded by non-infected endothelial cells but also by other cell types from the endothelium-underlying tissue, such as fibrocytes. To assess the principle capacity of different cell types and different donor origin to support of parasite development, purified MCHC were transferred to cell layers of primary endothelial cells of bovine (BUVEC) and human (HUVEC) origin, in addition to human fibrocytes (HFF). In principle, all different cell types (BUVEC, HUVEC and HFF) allowed MCHC integration and supported parasite development and merozoite I synthesis (**Figure 3A**). Surprisingly, the development of large macromeronts was best supported by HFF cells, followed by HUVEC and least by BUVEC (**Figure 3B**). Moreover, merozoite I synthesis was best supported by HUVEC and HFF, followed by BUVEC (**Figure 3C**), allowing for the assumption, that the overall best support came from human fibrocytes (HFF) and thereby from a cell type different from the classical host cell (bovine endothelial cells). However, this finding may reflect the situation *in vivo*, as already mentioned above. Moreover, the host origin of BCs did not seem to matter since human cells also supported macromeront formation.

When transferring MCHCs into cell-free culture dishes, representing meront-only-controls, some residual BCs regrew in the plates over time (experimental period of experiments 11–12 days), thereby giving rise to a new BUVEC layer over time serving as suitable BCs. Accordingly, both the macromeront size and merozoite I production in the latter experimental condition did not differ significantly from recipient BUVEC layers, but indeed showed a significant difference when compared to HFF (macromeront size: *p* = 0.0357 merozoite I production: *p* = 0.0303) and HUVEC (macromeront size: *p* = 0.0167, merozoite I production: *p* = 0.0303) (**Figures 3B, C**), which both supported macromeront formation in an improved manner.

### Non-infected BUVECs transfer mitochondria via TNT structures

**Figure 4:**
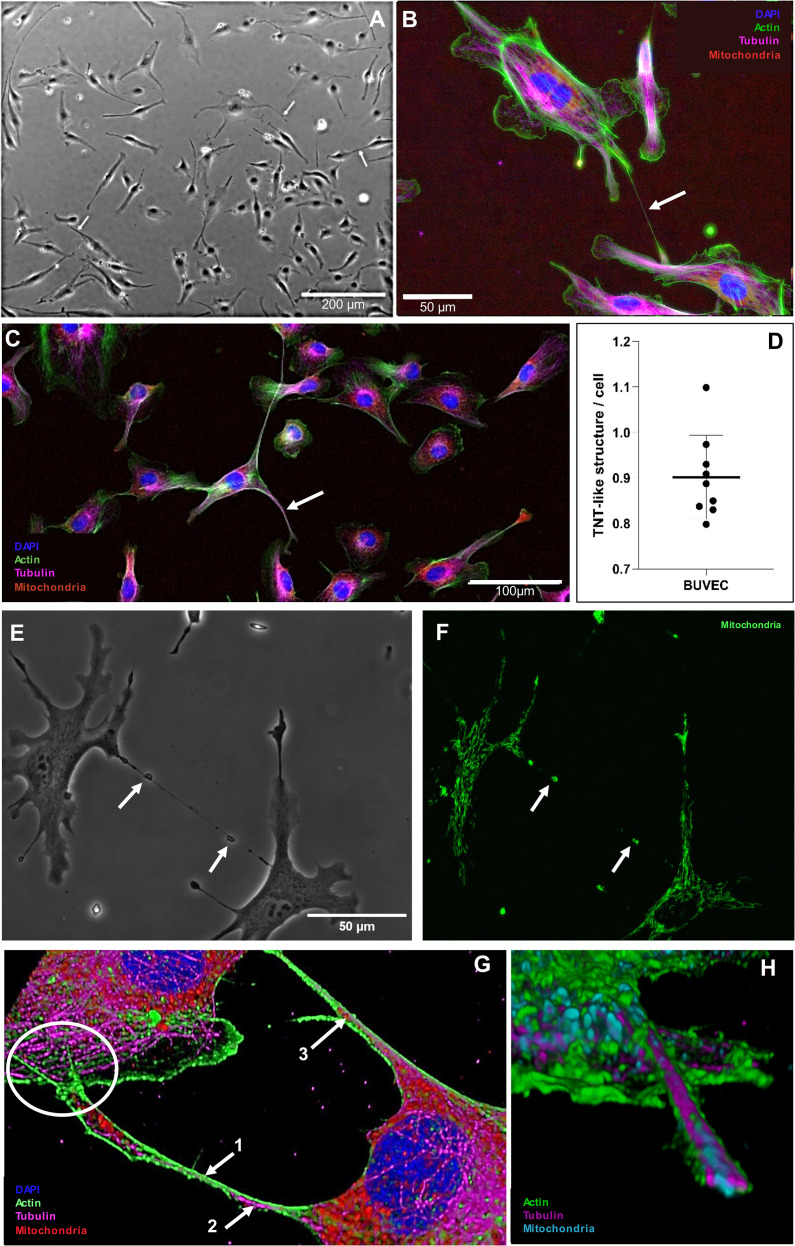
Formation of TNT-like structures by primary BUVEC. **(A)** TNT-like structure in phase contrast being formed by a single BUVEC (white arrow). **(B)** Subconfluent BUVEC stained with phalloidin CF488A (actin), anti-α tubulin (microtubules), anti-AIF (mitochondria) and DAPI (nucleus) highlighting the actin backbone of thin (500 nm) TNTs (white arrow). **(C)** Subconfluent BUVEC stained with phalloidin CF488A (actin), anti-α tubulin (microtubules), anti-AIF (mitochondria) and DAPI (nucleus) showing the presence of microtubules inside a thick (1900 nm) TNT (white arrow). **(D)** Quantification of TNT formation by non-infected BUVEC within 48 h of culture. Arithmetic mean and standard deviation of nine biological replicates. **(E)** Phase contrast and **(F)** mitochondrial staining of a thin TNT formed between two single BUVEC. Note microscopically visible bulges in phase contrast indicating the presence of mitochondria (white arrows) within the TNT. **(G)** 3D illustration of TNTs formed between two BUVECs. The TNT is composed of an actin (1. arrow) backbone with integrated microtubule elements (2. arrow). Inside the TNT, mitochondrial cargo (3. arrow) is visible. The current TNT has a diameter of 600 nm at the thinnest part and hovers over the connected cell (white circle) **(H)** 3D cross section of a TNT structure illustrating its composition [actin (green), tubulin (magenta) and mitochondria (blue)].

As recently described, intracellular *E. bovis*-macromeront development is an energy- and building block-demanding process, which, in consequence, drives host cells into premature senescence (Velásquez et al., 2021a). Interestingly, senescent cells were recently described to be “rescued” by other healthy cells via TNT-based mitochondria donation (Liu et al., 2014), thereby aiding them to return into a normal operating state. Following the hypothesis that TNTs may be involved in BC-based metabolic support of MCHCs, we here examined in a first series of experiments the principle capacity of primary non-infected BUVEC to form TNTs and to transfer mitochondria via TNTs. Therefore, different BUVEC isolates (*n* = 9) were cultured to subconfluency and TNT formation was microscopically estimated according to Tishchenko et al. (2020), applying the following TNT characteristics: straight membrane protrusions and a diameter of 70–4000 nm. In the non-infected control setting (**Figure 4A**), BUVEC formed 0.9 TNT-like structures per cell in average (**Figure 4D**). When staining TNT-like structures for actin, tubulin and mitochondria, we demonstrated that newly formed TNTs indeed showed an actin-based backbone and occasionally contained tubulin elements (**Figures 4B, C**), as indicative for thin and thick TNTs, respectively. Moreover, when merging phase contrast images with mitochondria staining, we showed that non-infected BUVEC indeed exchanged mitochondria (please note phase contrast-visible membrane bulges indicating the presence of mitochondria, **Figures 4E, F**), a phenomenon that was also confirmed by 3D rendering of confocal microscopic images (**Figures 4G, H**). Here, a TNT cross-section illustrated an outer layer of actin skeleton, inner microtubule elements and mitochondrial cargo (**Figure 4H**). Moreover, the hovering of a TNT above the substrate and the connecting cell was confirmed (**Figure 4G**), which represents a typical characteristic of TNTs that distinguishes these structures from other membranous protrusions like filopodia.

### *Eimeria bovis* infection boosts TNT formation

**Figure 5:**
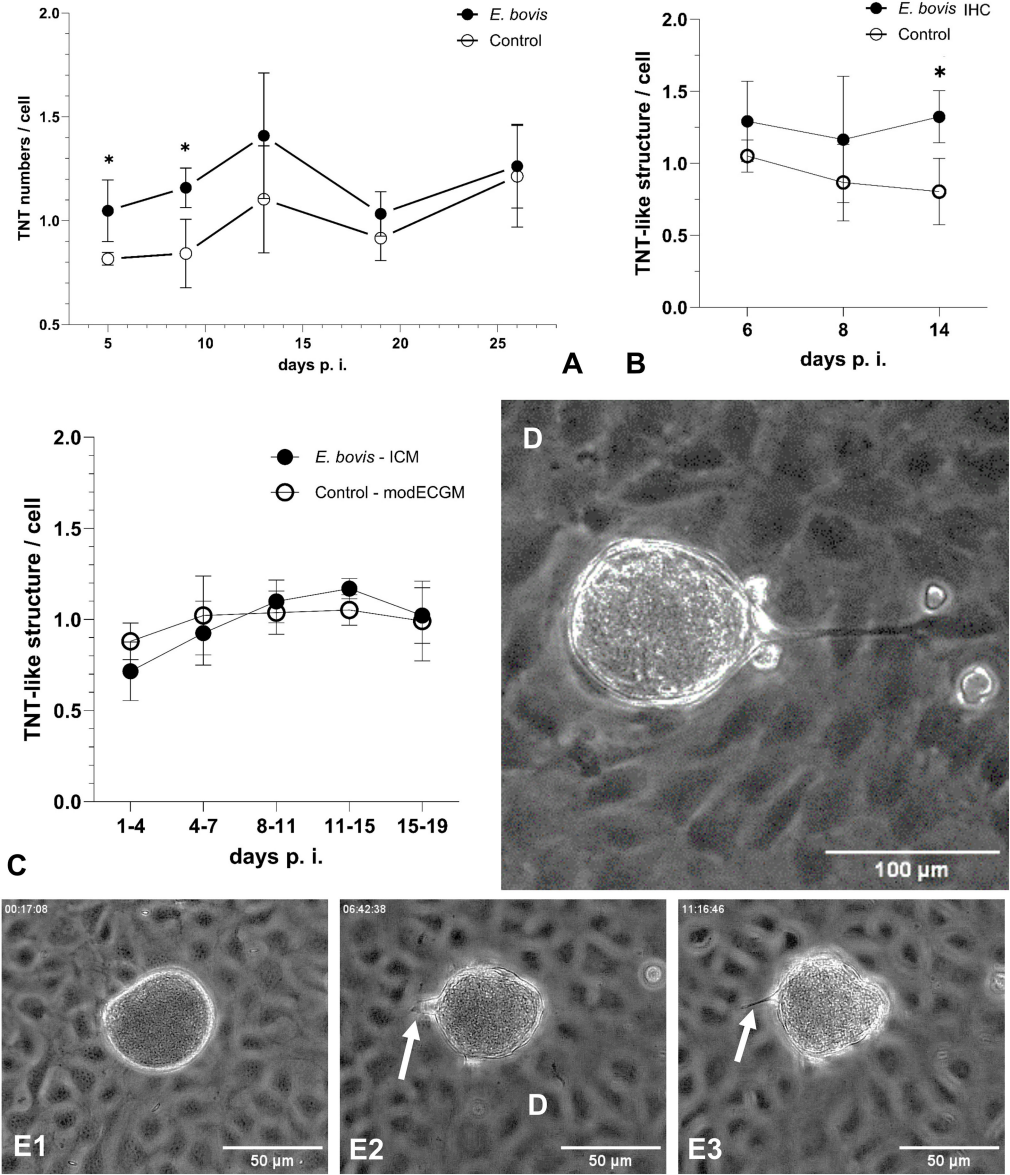
Formation of TNT-like structures by BUVEC during *E*. *bovis* macromeront development. **(A)** Kinetic of TNT formation in infected and non-infected cells within an *E*. *bovis*-infected BUVEC layer and of non-infected cells in control layers. **(B)** Quantification of TNT-like structures in single *E. bovis-*infected host cells at the early phase of *E*. *bovis* meront I formation. **(C)** Quantification of TNT-like structures in subconfluent BUVECs treated with infection-conditioned medium (ICM) or control medium. **(D)** Illustration of a MCHC-derived TNT hovering over BCs and connecting a distant non-infected BUVEC. **(E1–E3)** Kinetic of TNT formation between *E. bovis* MCHC and BC (white arrows) over 11 h. Arithmetic mean and standard deviation of four biological replicates; *p ≤ 0.05.

To test the hypothesis that intracellular parasite infection may drive TNT formation in host cells, we analysed subconfluent *E. bovis*-infected BUVEC layers (these contain both, infected and non-infected cells; *n* = 4) for TNT formation throughout macromeront development (5–26 days p. i.) using phase contrast microscopy, comparing them to non-infected control layers (**Figure 5**). Indeed, the number of TNT-like structures was upregulated in cells of *E. bovis*-infected cell layers by 28%, 38%, 28%, 11% and 4% at days 5, 9, 13, 19 and 26 p. i., respectively, reaching statistical significance at days 5 and 9 p. i. (5 days p. i.: p = 0.0286, 9 days p. i.: p = 0.0286; **Figure 5A**). These data indicated that TNT formation is especially induced in the early phase of meront formation, when the parasite prepares the host cell for its proliferation (in general, the first boost in meront size is observed around 7–8 days p. i.). When analysing single *E. bovis-*infected host cells in this early time frame, an upregulation of TNT formation was confirmed since the number of TNT-like structures was enhanced by 22%, 34% and 65% at days 4, 8 and 14 p. i., respectively, reaching statistical significance at day 14 p. i. (p = 0.0286; **Figure 5B**). These findings indicated that infected host cells themselves are more in need of cargo transfer at times of full proliferation (from 8 days p. i. onwards). Considering kinetics of TNT formation, we exemplary monitored a single *E. bovis*-infected host cells by live cell imaging and illustrated the formation of a single TNT within ~ 11 h (**Figures 5D, E**).

Given that non-infected cells within an infected BUVEC layer were also influenced in their TNT formation, secretory/excretory products of infected cells may have induced these effects. Therefore, we additionally tested infection-conditioned medium (ICM)-driven effects on TNT formation by analysing ICM-supplemented *E. bovis*-infected BUVEC layers. However, ICM treatments did not significantly affect TNT formation at any time point of macromeront development (**Figure 5C**).

### Inhibition of bystander cell-derived TNT formation impairs merozoite I production

**Figure 6:**
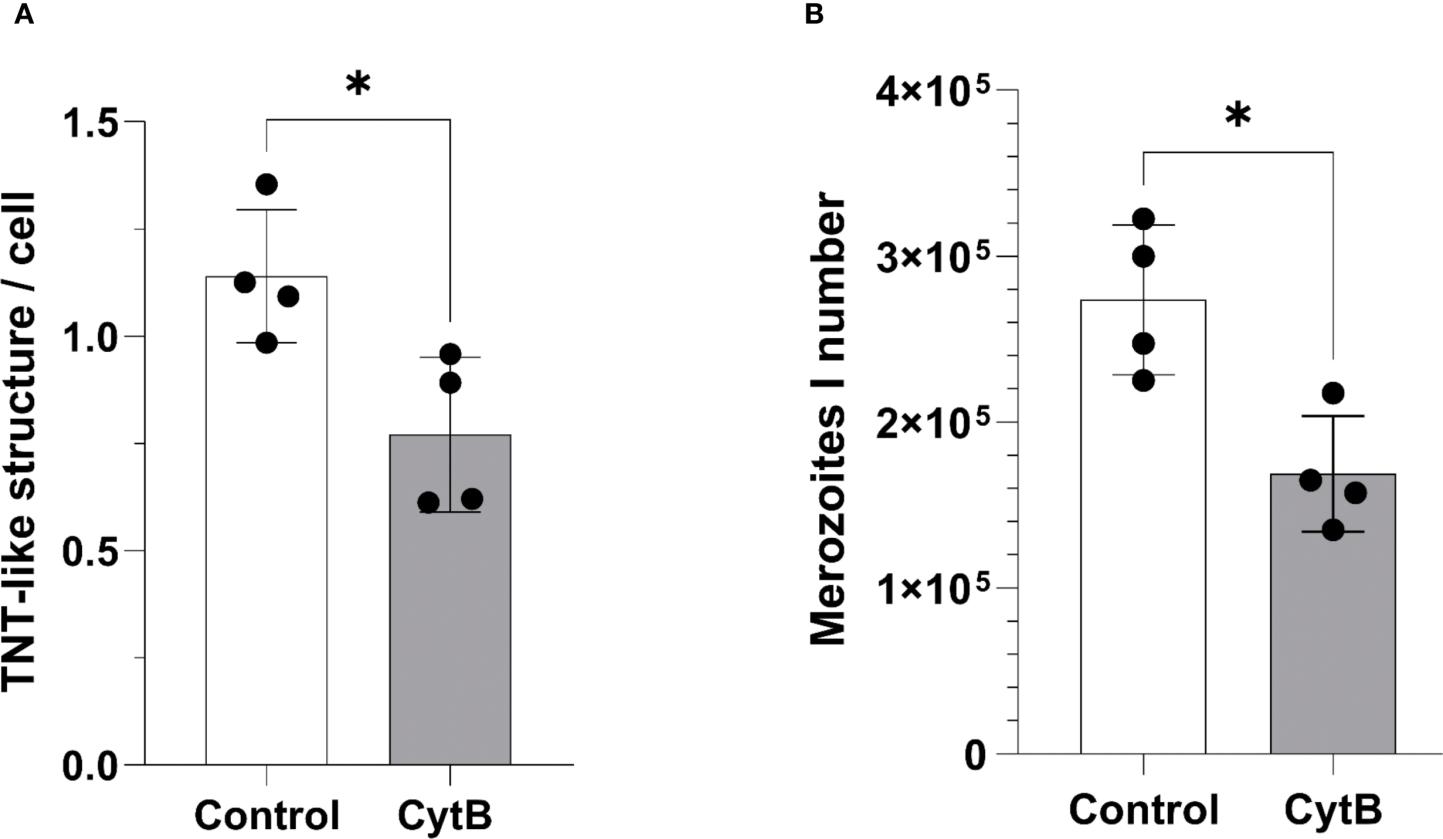
Effects of TNT inhibition on *E*. *bovis* macromeront development and merozoite I release. **(A)** Effects of low dose (350 nM) cytochalasin B treatments on TNT formation in non-infected BUVEC. **(B)** Effects of low dose (350 nM) cytochalasin B pretreatments of recipient BUVEC on merozoite I production after MCHC transfer. Arithmetic mean and standard deviation of four biological replicates; *p ≤ 0.05.

To assess the relevance of BC-derived TNT formation for effective *E. bovis* macromeront formation, we used the meront-transfer-system by pretreating recipient non-infected BUVEC (= BC) with inhibitors or stimulants of *de novo* TNT biogenesis. Unfortunately, selective inhibitors of TNT formation are currently lacking. To achieve an inhibition of TNT formation, we here applied the method described by Bukoreshtliev et al. (2009), who developed a protocol to selectively block TNT biogenesis by nanomolar cytochalasin B concentrations, which did not affect endo- and phagocytosis in PC12 cells but effectively inhibited intercellular organelle transfer. To furthermore exclude direct effects of cytochalasin B treatments on *E. bovis* macromeront formation, which was previously described to rely on actin-based host cellular cytoskeletal rearrangements (Hermosilla et al., 2008), we applied the meront-transfer-system and exclusively treated recipient non-infected BUVEC (= BC) with 350 nM cytochalasin B before transferring MCHCs. As expected, TNT formation was significantly (*p* = 0.0286) reduced by 38%, but not entirely blocked in cytochalasin B-treated non-infected BUVEC (**Figure 6A**). Moreover, when transferring non-treated MCHCs to cytochalasin-treated recipient BUVEC, macromeront development was indeed impaired since merozoite I production was significantly diminished when compared to non-treated recipient BUVEC (*p* = 0.0286, **Figure 6B**). These data underlined the relevance of BC-derived TNT formation for effective macromeront development.

### Bidirectional transfer of mitochondrial cargo between BUVEC and *Eimeria bovis*-infected host cells via TNT

**Figure 7:**
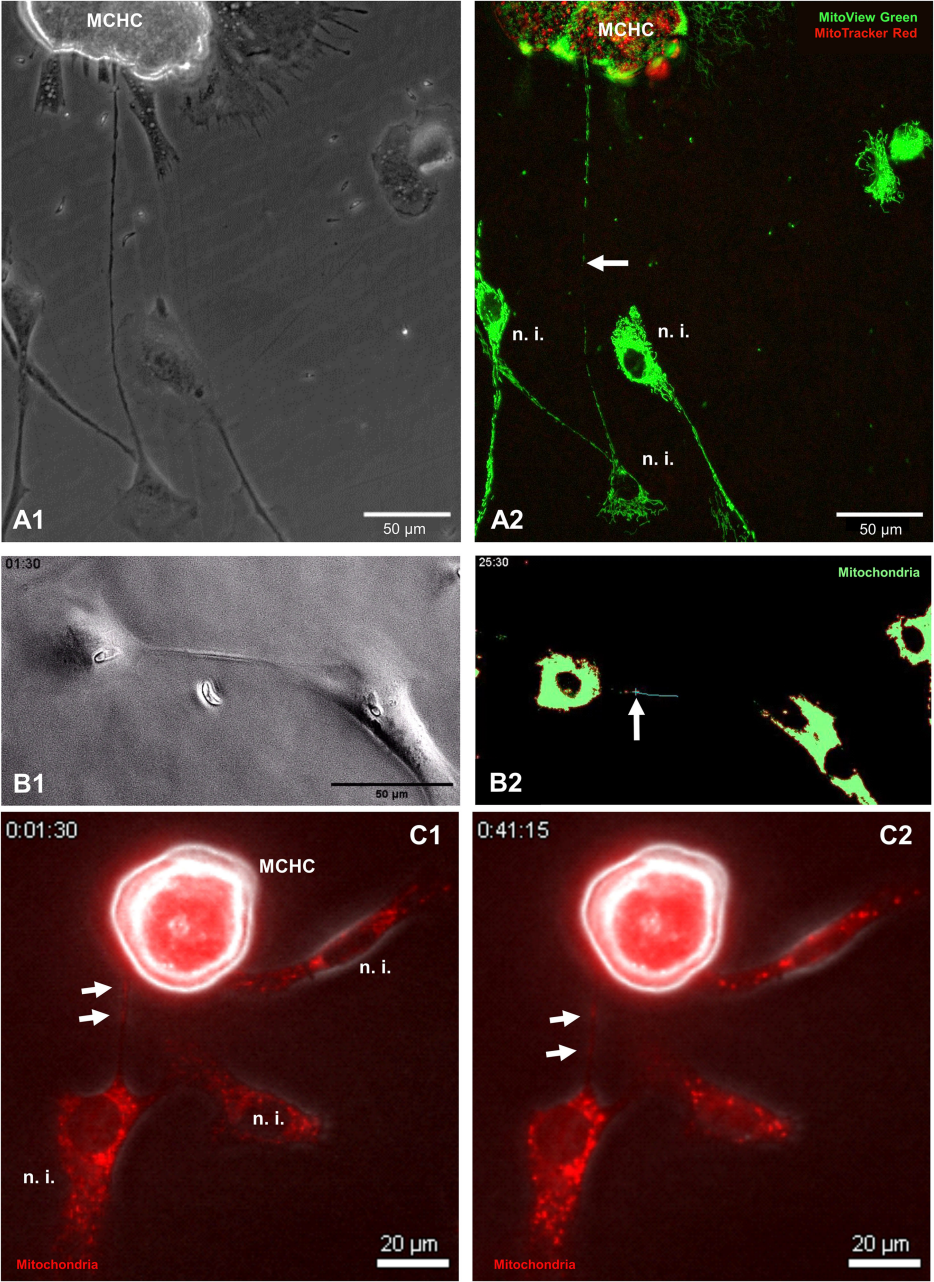
Illustration of TNT-based mitochondria transfer between MCHC and non-infected BUVEC. **(A1, A2)** Phase contrast **(A1)** and fluorescence-based illustration **(A2)** of MCHC-derived mitochondria (MitoTracker™, RedCMXRos-stained) and non-infected BUVEC-derived mitochondria (MitoView™ Green-stained) at 19 days p. i. Note the TNT-based connection of MCHC and non-infected BUVEC shuttling green (non-infected BUVEC-derived) mitochondria (white arrow). **(B1, B2)** Phase contrast **(B1)** and fluorescence-based [MitoView™ Green-stained, **(B2)**] illustration of two *E. bovis* sporozoite-infected BUVECs (4 days p. i.) connected via a single TNT, in which a mitochondrion (green staining) is transferred over a period of 24 minutes. The mitochondrial cargo (marked by a blue cross) moves with 17 nm/sec = 1.02 µm/min within the TNT. **(C1, C2)** Phase contrast and fluorescence merge (MitoTracker Red-stained) of MCHC (15 days p. i.) connected to a non-infected BUVEC via TNT. The mitochondrial cargo (white arrows) is transferred by the MCHC to a recipient non-infected BUVEC (time laps over 45 minutes).

Given that BC-derived TNT formation seemed relevant for *E. bovis* macromeront formation, we next analysed whether mitochondria are transferred via TNTs to support the energetic status of *E. bovis*-infected host cells, which were previously shown to experience infection-driven dysregulation of mitochondrial function (Velásquez et al., 2021b). To analyse the principle occurrence and direction of TNT-based mitochondria exchange between *E. bovis*-infected and non-infected cells, we applied the meront-transfer-system. Therefore, mitochondria of MCHCs (isolated at day 21 p. i.) were first labelled in red with the live dye MitoTracker Red and then transferred to subconfluent non-infected BUVEC, which had previously been stained for mitochondria in green by the live dye MitoView Green. One day after co-culture, TNT-based mitochondria transfer was analysed by fluorescence microscopy simultaneously using green and red channels for mitochondria illustration. As illustrated in **Figure 7A**, TNT-based mitochondria transfer from a non-infected cell to a MCHC was unveiled. In an exemplary case, a MCHC (22 days p. i.) was connected via a long TNT (≈ 250 µm) to a single distant BUVEC while hovering over another non-infected cell (**Figure 7A**). Given that we only found green mitochondria (source: non-infected BUVEC) to be transferred in this co-culture, cargo exchange seemed mainly unidirectional. Interestingly, the donating non-infected cells was not only connected by a TNT to the MCHC, but also to other non-infected cell and showed a weaker fluorescence intensity than other non-infected cells, which may potentially reflect its reduced mitochondrial content (**Figure 7A**). Additionally, we also illustrated a discard of mitochondria from MitoTracker Red-stained MCHC to non-infected BUVEC via live cell imaging over 45 min (**Figure 7C**). So far, these observations merely represent a proof of concept, demanding for further detailed analyses on the precise numbers, direction or speed of mitochondria transfer. However, when exemplarily monitoring the TNT-based exchange of MitoView Green-stained mitochondria between two *E. bovis* sporozoite-infected cells at 4 days p. i. by live cell imaging, the speed of transfer accounted to 0.017 μm/s, which is slower than the average transport speed of mitochondria described in axons (Niescier et al., 2016) (**Figure 7B**).

## Discussion

As a typical apicomplexan parasite, *E. bovis* successfully controls its host cell to guarantee its obligate intracellular development. Hence, *E. bovis* was recently shown to efficiently alter host cell apoptosis (Lang et al., 2009), cytoskeletal arrangement (Hermosilla et al., 2008), endothelium-derived innate cellular defence mechanisms (Hermosilla et al., 2015) and host cellular metabolism (Hamid et al., 2014, 2015; Velásquez et al., 2021b, 2021a). Moreover, during merogony I, host endothelial cells carrying *E. bovis* macromeronts experience significant metabolic stress finally resulting in premature senescence (Velásquez et al., 2021a). Under physiological conditions, such a state would typically lead to cell death. In case of *E. bovis* MCHCs, these infected cells must survive for ~ 2–3 weeks until merozoite I release *in vivo*. To date, it remains unclear, how a single host endothelial cell can deliver sufficient energy and building blocks to support the generation of > 140,000 merozoites I. Hence, it is tempting to hypothesize that *E. bovis*-infected cells may be aided by other cells from the tissue environment. In this context, we here confirmed an accumulation of neighbouring BCs around *E. bovis-*infected host cells, which was boosted with ongoing macromeront maturation. Obviously, an enhanced number of BCs at lateral sides of infected cells may also be attributed to the increasing size of MCHCs or to an enhanced confluency of cell layers throughout the cultivation period of 3 weeks, even though endothelial cells generally cease proliferation when confluency is achieved (Eagle and Levine, 1967; Viñals and Pouysségur, 1999; Pavel et al., 2018) In line, non-infected primary BUVEC did not show BC accumulation in a confluent monolayer in the current experimental setting. Moreover, even in highly confluent BUVEC layers, we never observed cells growing below or on top of other cells as it was the case for *E. bovis*-infected BUVEC layers. Therefore, we hypothesize that BC accumulation is a parasite-driven phenomenon, which may reflect a supporting system for MCHCs accomplished by neighbouring cells. Considering *E. bovis* macromeront’s needs for sterols and energy, which rises with ongoing maturation (Hamid et al., 2015; Taubert et al., 2018; Velásquez et al., 2021b; Silva et al., 2022), it seems opportune that BC numbers increase with ongoing infection time. Being strictly host- and cell type-specific, macromeronts exclusively develop in bovine endothelial cells (Hermosilla et al., 2002, 2012). Obviously, *in vivo*, host cells are not only in contact with neighbouring endothelial cells but also with other cell types of the underlying tissue, such as fibrocytes. Of note, even though BUVEC allow full macromeront formation *in vitro*, they do not support equal macromeront sizes as seen *in vivo* (Hammond et al., 1946). Interestingly, infections of a primary bovine fetal gastrointestinal cell (BFGC) line containing both epithelial and endothelial cells lead to the formation of larger macromeronts with higher merozoite I production than BUVEC (Hermosilla et al., 2002). To test the hypothesis that efficient macromeront formation in BUVEC relies on external support by other (non-infected) cells, we here studied if MCHC transfer to other primary cells would also support *E. bovis* macromeront maturation. In line with the physiological tissue setting, *E. bovis* macromeront growth was indeed best supported by HFF cells, i. e. by a cell type, which is frequently used as potent feeder cell within multi-cell type systems (Pekkanen-Mattila et al., 2012). Of note, whilst *E. bovis* sporozoites fail to develop further in any cell type of human or primate origin (Hermosilla et al., 2002) or in fibroblasts, HUVEC and HFF both better supported macromeront development when acting as BCs than BUVEC. BUVEC, in turn, represents the only of these cell types allowing for parasite development, but seems of minor capacity in serving as supporter cells.

Obviously, intercellular communication and molecule transfer represent a prerequisite for MCHC-BC interactions. Besides other mechanisms like gap junction-based rapid transfer of ions and metabolites (Evans and Martin, 2002), many cell types were meanwhile described to exchange all kinds of cargo via TNTs. TNTs are thin, straight and long-reaching membranous protrusions that connect spatially separated cells with another (Rustom et al., 2004; Vignais et al., 2017; Drab et al., 2019; Jansens et al., 2020; Cervantes and Zurzolo, 2021; Dagar et al., 2021). Unlike substrate-adherent peripheral filopodia, TNTs hover at upper planes over the substrate or even over other cells. Typically, TNTs are rich in actin and eventually also contain tubulin elements, and act as conduits for intercellular exchange of a plethora of cargo, such as cytosolic proteins, ions, miRNAs and organelles like endoplasmic reticulum, Golgi vesicles, endosomes, lysosomes, lipid droplets or mitochondria (Rustom et al., 2004; Austefjord et al., 2014; Drab et al., 2019; Jansens et al., 2020; Dagar et al., 2021), with the two latter ones being highly abundant or dysregulated, respectively, in MCHCs (Hamid et al., 2015; Silva et al., 2022). TNT formation mainly represents a typical feature of cells under stress and is boosted by various factors like serum or glucose concentration, viral infection or exposure to therapeutics (Vignais et al., 2017). Of note, TNT formation is triggered by low FCS conditions and enhanced ROS levels, both findings that parallel observations on boosted macromeront formation under low FCS (Taubert et al., 2018) and on increased ROS production in *E. bovis*-infected cells (Velásquez et al., 2021b), respectively. Even more interesting, it was shown that endothelial progenitor cells (EPC) transferred mitochondria via TNTs to revitalize senescent endothelial cells (Liu et al., 2014), thereby mirroring the adverse state that MCHCs experience at late merogony I (Velásquez et al., 2021a). Surprisingly, studies on the role of TNTs for intracellular parasite proliferation hardly exist. Hence, only one protozoa-related study reports that *Leishmania donovani* induces the formation of TNTs in B cells for their spread between cells (Stögerer et al., 2023), a phenomenon that was commonly reported for several viruses and bacteria (Kim et al., 2019; Jansens et al., 2020; Jahnke et al., 2022). Several obstacles hamper TNT detection: *i)* a TNT-specific marker is not yet available, *ii*) it is difficult to detect TNTs in dense/confluent cell layers or even in tissues and *iii)* based on their biological properties (thin + long), TNTs are fragile and vulnerable to light and shear forces resulting in breakage or bending (Koyanagi et al., 2005) when cells are fixed. To circumvent these obstacles, we here used sparse 2D cultures and mainly analysed TNTs by live cell imaging. Following this strategy, we first confirmed the general capacity of bovine endothelial cells (BUVEC) to form TNTs and detected a low TNT number in resting cells (0.9 TNTs/cell). We furthermore showed that *de novo* TNT biogenesis can be inhibited by low dose cytochalasin B treatments (reduction to 62% TNTs/cell). By combining phase contrast, fluorescence and live cell microscopy, we illustrated BUVEC to generate genuine TNTs, connecting two cells while hovering over the substrate and showed that these structures own an actin-based backbone and harbour tubulin elements. Of note, TNTs rapidly build up, dislodge and break down (Sowinski et al., 2008), consequently, each data set just reflects a snapshot of dynamic TNT biogenesis. Assuming that BCs support *E. bovis*-infected host cells via TNT-based molecule or organelle donation, we furthermore analysed TNT formation of MCHCs throughout macromeront formation *in vitro*. Applying above mentioned criteria, current data confirmed a significant, parasite-driven increase in TNT numbers of up to 38% by cells within infected BUVEC layers (comprising both non-infected and infected cells) and up to 65% in *E. bovis*-infected host cells themselves. The finding, that *E. bovis* infection led to enhanced TNT formation also in non-infected cells within an infected cell layer, pointed at potential effects of soluble molecules being excreted/secreted by infected cells. However, treatments of BUVEC with infection-conditioned medium did not change TNT formation and therefore did not confirm this assumption but potentially requires MCHC to be present.

The key role of BC-derived TNT formation for successful *E. bovis* macromeront formation was underlined by TNT inhibition experiments. Given that specific inhibitors of TNT formation are currently unavailable, we here used nanomolar treatments of cytochalasin B [a compound that inhibits actin polymerization (MacLean-Fletcher and Pollard, 1980)], which were recently shown to selectively block TNT-based organelle transfer but not to affect other actin-based cellular functions like endo- or phagocytosis in PC12 cells (Bukoreshtliev et al., 2009). In our hands, low dose cytochalasin B (350 nM) treatments of non-infected BUVEC reduced cell proliferation but did not affect cell viability. Importantly, BUVEC-derived TNT formation was diminished but not entirely blocked by cytochalasin B treatments. Since *E. bovis* macromeront formation was previously proven to be accompanied by a significant host cellular actin cytoskeleton rearrangement (Hermosilla et al., 2008), we abstained from treating *E. bovis*-infected cells and exclusively treated non-infected recipient cells before adding MCHCs via the meront-transfer-system. As expected, cytochalasin B treatments of meront-recipient BUVEC indeed diminished merozoite I production, thereby might confirming a role of BC-derived TNTs or the BC cytoskeleton for MCHC support. By definition, TNTs are thin membrane protrusions that connect distant cells, hover in an upper optical plane, contain actin and transport intercellular cargo (Cervantes and Zurzolo, 2021). To also verify the latter characteristics and to address why BC-based TNT formation is beneficial for MCHCs, we next analysed if mitochondria, representing the powerhouses of cells, are transferred from BCs via TNTs to MCHCs. As recently described, TNT-based transfer of healthy mitochondria leads to their incorporation into the endogenous network of recipient cells, thereby triggering bioenergetic changes, which may allow recipient cells to recover from pathological processes and to return to a normal operating state (Luchetti et al., 2022). Given that *E. bovis*-infected host cells were demonstrated to experience mitochondrial dysfunction (Velásquez et al., 2021b), healthy mitochondria donation by BCs may aid infected cells to recover enabling them to promote macromeront formation. In fact, when applying differential staining on MCHC- and BC-derived mitochondria, a transfer of BC-derived mitochondria to *E. bovis*-infected host cells was illustrated. Here, a mitochondrial transfer speed of 0.017 µm/s was measured, which is on the lower end of mitochondrial velocity in axons, but still reasonable (Niescier et al., 2016). Notably, while most studies reported the transfer of functional mitochondria to damaged cells for cell healing, this process is not obligatory unidirectional since damaged mitochondria may also be transferred to healthy cells (Mahrouf-Yorgov et al., 2017). Consequently, MCHCs may also profit from TNT-based exchange by discarding defect mitochondria to healthy BCs to balance their mitochondrial dysfunction state (Velásquez et al., 2021b) as demonstrated in the current work. Hence, future experimentation is needed to thoroughly study in depth the extent and direction of mitochondrial exchange between MCHCs and BCs.

Overall, with the current report, we deliver a comprehensive set of data, which will contribute to our understanding of not only *E. bovis* biology, suggesting a new multicellular system of intracellular parasite support, but also for other pathogenic ruminant macromeront forming *Eimeria* species like *E. zuernii* (cattle), *E. arloingi*, *E. ninakohlyakimovae* (goats) and *E. ovinoidalis* (sheep). Further investigations will particularly address the underlying molecular mechanisms in addition to the extent and direction of exchange of different cargos between BCs and MCHCs.

## Data Availability

The original contributions presented in the study are included in the article/**Supplementary Material**. Further inquiries can be directed to the corresponding authors.
