# Supplementary material for: Intracellular Eimeria bovis macromeront formation induces bystander cell accumulation and TNT formation
Source: Front Cell Infect Microbiol. 2025 Sep 26;15:1665269. doi: 10.3389/fcimb.2025.1665269 (PMC12531633; doi:10.3389/fcimb.2025.1665269)
Supplement: Supplementary file 1 [file DataSheet1.pdf]

Viability assays

Cell toxicity of the chemical compound cytochalasin B was tested by using SYTOX™Orange (Thermo Fisher Scientific, Waltham, MA, USA). Therefore, BUVEC were treated with cytochalasin B for 24 h (350 nM). Thereafter, BUVEC were stained by SYTOX™Orange (5 µM, 5 min, RT), which only penetrates dying or dead cells. For negative and positive controls, non-treated and Triton X-treated BUVEC were used, respectively. The percentage of viable cells was calculated in randomly taken microscopic images. Overall, treatments with H89 and Y26732 showed no significant cell toxicity in the range of 0.5 – 64 µM for Y26732 and 0.5 – 32 µM H89 (Fig. 1 A-B). Moreover, BUVEC treated with 350 nM cytochalasin B exhibited no cell death (> 99.9 % of BUVEC viable) after 24 h (Fig. 1 A).

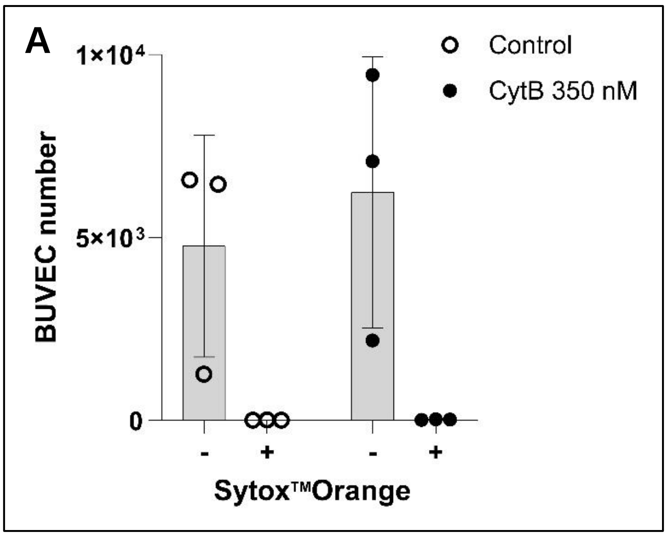

Fig. 1 Cell toxicity assays for Cytochalasin B used in experimental setting **(A)** Quantification of viable and dead (Sytox™Orange) cells of cytochalasin B treated BUVEC after 24 h

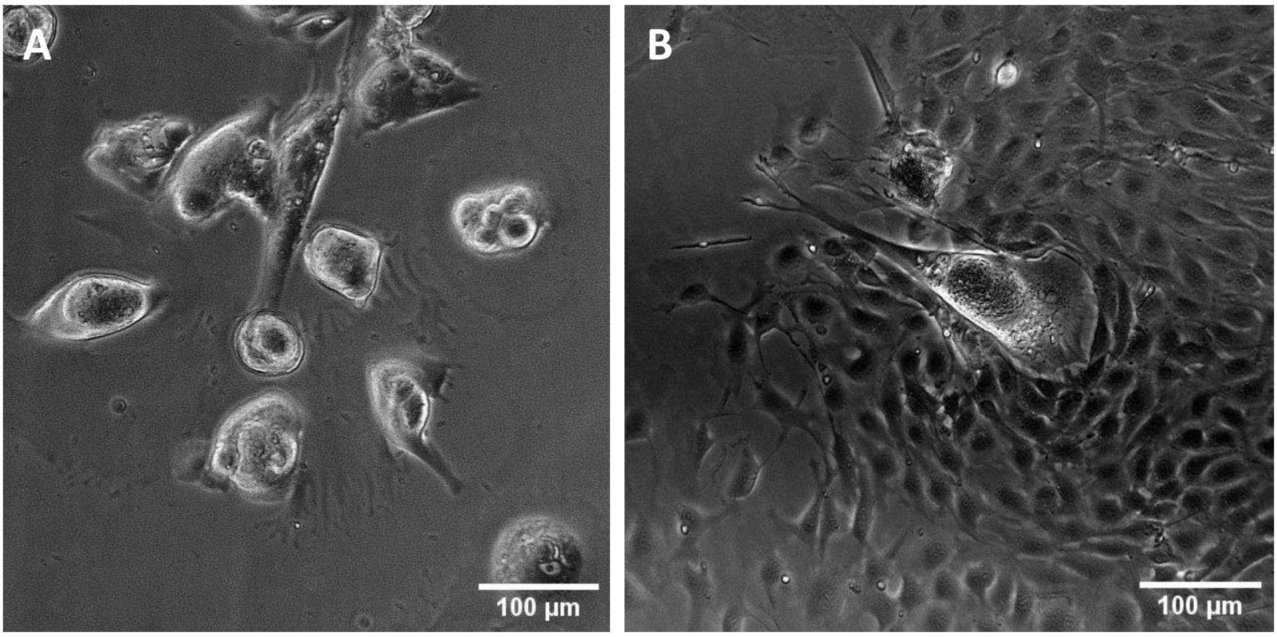

Suppl. Fig. 3. Only meronts

Exemplary illustration of MCHCs transferred to wells without a recipient cell layer at **(A)** 1 day p. t. and **(B)** 8 days p. t., showing the regrowth of BCs over time. On the right side, residual gaps are still visible, reflecting areas where BCs are repopulate the space.
